# Supplementary material for: A Critical View of the Application of the APEX Software (Aqueous Photochemistry of Environmentally-Occurring Xenobiotics) to Predict Photoreaction Kinetics in Surface Freshwaters
Source: Molecules. 2019 Dec 18;25(1):9. doi: 10.3390/molecules25010009 (PMC7017383; doi:10.3390/molecules25010009)
Supplement: Supplementary file 1 [file molecules-25-00009-s001.zip › 3.2.4_gcc-4.4.0.zip!/3.2.4_gcc-4.4.0/3.2.4_gcc-4.4.0/doc/octave/html/interpreter/Built_002din-Data-Types.html]

Built-in Data Types - Untitled


Next: User-defined Data Types,
Up: Data Types


---

### 3.1 Built-in Data Types

The standard built-in data types are real and complex scalars and
matrices, ranges, character strings, a data structure type, and cell
arrays. Additional built-in data types may be added in future versions.
If you need a specialized data type that is not currently provided as a
built-in type, you are encouraged to write your own user-defined data
type and contribute it for distribution in a future release of Octave.

The data type of a variable can be determined and changed through the
use of the following functions.

— Built-in Function: **class** (expr)  
— Built-in Function: **class** (s, id)  
— Built-in Function: **class** (s, id, p, ...)  
> Return the class of the expression expr or create a class with
> fields from structure s and name (string) id. Additional
> arguments name a list of parent classes from which the new class is
> derived.

— Function File: **isa** (x, class)  
> Return true if x is a value from the class class.

— Function File: **cast** (val, type)  
> Convert val to data type type.
>
>
>
> **See also:** int8, uint8, int16, uint16, int32, uint32, int64, uint64, double.

— Loadable Function: **typecast** (x, type)  
> Convert from one datatype to another without changing the underlying
> data. The argument type defines the type of the return argument
> and must be one of 'uint8', 'uint16', 'uint32', 'uint64', 'int8', 'int16',
> 'int32', 'int64', 'single' or 'double'.
>
> An example of the use of typecast on a little-endian machine is
>
> ```
>           x = uint16 ([1, 65535]);
>           typecast (x, 'uint8')
>           => [   0,   1, 255, 255]
> ```
>
>
>
>
> **See also:** cast, swapbytes.

— Function File: **swapbytes** (x)  
> Swaps the byte order on values, converting from little endian to big
> endian and vice versa. For example
>
> ```
>           swapbytes (uint16 (1:4))
>           => [   256   512   768  1024]
> ```
>
>
>
>
> **See also:** typecast, cast.

- Numeric Objects- Missing Data- String Objects- Data Structure Objects- Cell Array Objects
